# The erythropoietin-derived peptide MK-X and erythropoietin have neuroprotective effects against ischemic brain damage

**DOI:** 10.1038/cddis.2017.381

**Published:** 2017-08-17

**Authors:** Seung-Jun Yoo, Bongki Cho, Deokho Lee, Gowoon Son, Yeong-Bae Lee, Hyung Soo Han, Eunjoo Kim, Chanil Moon, Cheil Moon

**Affiliations:** 1 Department of Brain & Cognitive Sciences, Graduate School, Daegu Gyeongbuk Institute of Science and Technology (DGIST), Daegu 711-873, Republic of Korea; 2 Convergence Research Advanced Centre for Olfaction, Daegu Gyeungbuk Institute of Science and Technology, Daegu, Korea; 3 Department of Neurology, Gil Medical Center, Gachon University, Incheon 405-760, Republic of Korea; 4 Department of Physiology, School of Medicine, Kyungpook National University, Daegu 700-422, Republic of Korea; 5 Division of Nano and Energy Convergence Research, Daegu Gyeongbuk Institute of Science and Technology (DGIST), Daegu 711-873, Republic of Korea; 6 GemVax, Unjun-ro, Bundang Seongnam-si, Gyeonggi-do 13467, Republic of Korea

## Abstract

Erythropoietin (EPO) has been well known as a hematopoietic cytokine over the past decades. However, recent reports have demonstrated that EPO plays a neuroprotective role in the central nervous system, and EPO has been considered as a therapeutic target in neurodegenerative diseases such as ischemic stroke. Despite the neuroprotective effect of EPO, clinical trials have shown its unexpected side effects, including undesirable proliferative effects such as erythropoiesis and tumor growth. Therefore, the development of EPO analogs that would confer neuroprotection without adverse effects has been attempted. In this study, we examined the potential of a novel EPO-based short peptide, MK-X, as a novel drug for stroke treatment in comparison with EPO. We found that MK-X administration with reperfusion dramatically reduced brain injury in an *in vivo* mouse model of ischemic stroke induced by middle cerebral artery occlusion, whereas EPO had little effect. Similar to EPO, MK-X efficiently ameliorated mitochondrial dysfunction followed by neuronal death caused by glutamate-induced oxidative stress in cultured neurons. Consistent with this effect, MK-X significantly decreased caspase-3 cleavage and nuclear translocation of apoptosis-inducing factor induced by glutamate. MK-X completely mimicked the effect of EPO on multiple activation of JAK2 and its downstream PI3K/AKT and ERK1/2 signaling pathways, and this signaling process was involved in the neuroprotective effect of MK-X. Furthermore, MK-X and EPO induced similar changes in the gene expression patterns under glutamate-induced excitotoxicity. Interestingly, the most significant difference between MK-X and EPO was that MK-X better penetrated into the brain across the brain–blood barrier than did EPO. In conclusion, we suggest that MK-X might be used as a novel drug for protection from brain injury caused by ischemic stroke, which penetrates into the brain faster in comparison with EPO, even though MK-X and EPO have similar protective effects against excitotoxicity.

Stroke is a common disease in adults and remains a leading cause of death and hospitalization and the biggest driver of prescription drug use. Ischemic stroke caused by breakdown of blood supply to the brain is the most common type (~85%) of stroke.^1, 2^ Despite recovery of blood supply by reperfusion, massive excitotoxicity-mediated neuronal death occurs.^3^ The excitotoxicity results mainly from hyperactivation of NMDA receptor by excessive release of glutamate during ischemia, which leads to intracellular calcium overload and oxidative stress induced by reactive oxygen species (ROS), resulting in mitochondrial dysfunction and membrane permeabilization.^4^ Eventually, cytochrome *c* and apoptosis-inducing factor (AIF) are released from the mitochondrial intermembrane space, which leads to caspase-dependent and -independent neuronal death.^5^ Therefore, pharmacological administration to reduce excitotoxicity has been considered critical for treatment of ischemic stroke in addition to surgical reperfusion.^6^ Unfortunately, although some inhibitors of glutamate release, NMDA receptor, and cell death pathway have been proposed, most clinical trials have failed because of low efficiency and side effects.^7^ Therefore, neuroprotection strategies for ischemic stroke still remain problematic.

Erythropoietin (EPO) is a well-known hematopoietic cytokine that promotes survival, proliferation, and differentiation of committed erythroid progenitor cells.^8^ Recently, many researchers became interested in non-hematopoietic functions of EPO, especially protection of cells in various organs (such as brain, retina, heart, and kidney) against ischemic injury.^9^ In the central nervous system, EPO is produced by neurons and astrocytes according to the oxygen status, and its receptor (EPOR) is also expressed throughout the developing and adult brain in mammals.^10^ During mid-gestation in mice, EPOR is highly expressed in the epithelium of the neural tube, where proliferating neuroprecursors reside, similar to that in the adult hematopoietic tissue.^11^ In adult brain, EPOR expression is elevated in the hippocampus, cortex, and midbrain.^12^ Identification of the spatial and temporal patterns of EPOR expression during brain development has improved our understanding of the potential range of EPO responses in the brain during ischemic/hypoxic stress or related neurological diseases, and provided insight into the utility of EPO treatments. In addition to the expression of EPO and EPOR in the brain, EPO possibly passes the blood–brain barrier (BBB) by receptor-mediated transcytosis.^13, 14^ Through this mechanism, peripherally administered EPO may cause various effects in the brain.

There may exist two distinct types of EPO receptors; ‘a classical homodimeric EPO receptor (EPOR)’ and ‘a cell protective EPO receptor (EPO-receptor)’. Administration of EPO induces autophosphorylation of Janus tyrosine kinase 2 (JAK2) by interaction of EPO to EPO-receptors, which subsequently phosphorylates and thus activates signal transducer and activator of transcription 5 (STAT5), finally leading to downstream transcriptional activation of target genes.^15^ Simultaneously, autophosphorylated JAK2 also phosphorylates EPO-receptors, resulting in activation of multiple secondary signaling molecules including mitogen-activated protein kinases (MAPKs) and phosphatidylinositol 3-kinase (PI3K).^8^ Because EPO-receptors activation has anti-apoptotic and antioxidant effects, EPO has been proposed as a putative drug target for neurodegenerative disease, especially in various animal models of ischemia.^16, 17^ Although how EPO ameliorates hypoxic–ischemic brain injury is still unclear, it is well documented that EPO has neuroprotective activity because it inhibits apoptosis and attenuates ROS generation by modulating superoxide dismutase and glutathione peroxidase.^18, 19^ Despite a strong neuroprotective effect of EPO, preclinical studies have exhibited limitations in its efficiency and safety.^20^ Administration of EPO in patients with anemia due to chronic kidney disease increases the risks of myocardial infarction, composite endpoints of death, and cardiovascular events. Even worse, EPO enhances tumor progression in patients with some types of cancer.^21, 22^ Therefore, more effective and delicate EPO-based strategies are required for treatment of ischemic stroke.

Previously, we designed MK-X, a novel short peptide (21 amino acids) derived from the EPO site that binds to the weak binding site of EPO-receptor, and found that MK-X treatment has the neuroprotective effect under oxidative stress without the proliferation effect.^23^ Here, we examined the potential of MK-X as a therapeutic agent in an ischemic stroke model.

## Results

### MK-X efficiently alleviates brain injury *in vivo* in a rat stroke model

**Figure 1:**
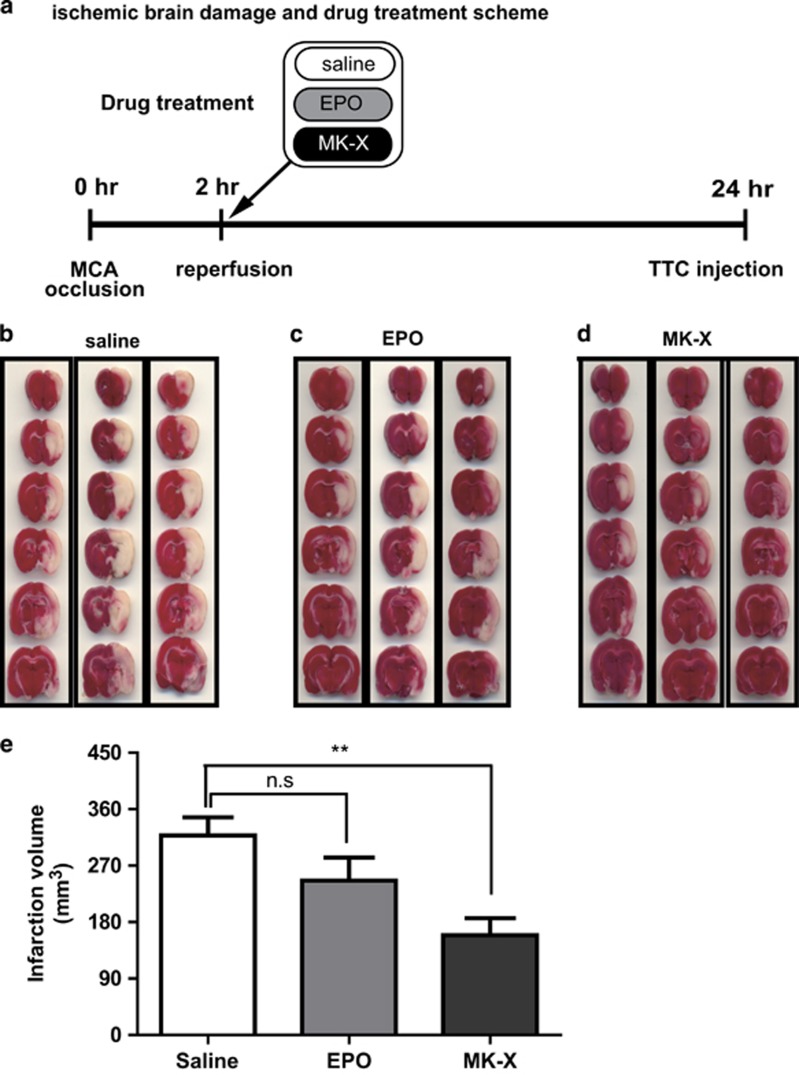
Effect of MK-X and EPO treatment on infarction volume 24 h after reperfusion. (**a**) Experimental scheme for the assessment of ischemic brain damage and the effects of MK-X and EPO treatments. (**b**–**d**) TTC staining of the brain. Non-ischemic areas appear red, and ischemic areas appear white. A pronounced decrease was observed in ischemic area in rats treated with MK-X but not EPO. (**e**) Quantitative analysis of cerebral infarct volume in each group (*n*=8 or 9). Determination of cerebral infarct volume by stereological analysis using the ImageJ program. For statistical analysis, one-way ANOVA was performed, followed by Dunnett’s *post hoc* test. Statistical significance is denoted (**P<0.01; n.s., non-significant)

We examined the potential of MK-X as a novel drug by using an *in vivo* ischemic stroke model. We induced transient ischemia by middle cerebral artery occlusion (MCAO) in rats for 2 h, and then performed reperfusion with or without EPO or MK-X treatment^24, 25^ (Figure 1a). After 24 h, we found that MCAO-reperfusion induced extensive infarction throughout all ipsilateral brain regions including the cerebral cortical and subcortical areas, but not in contralateral brain regions (Figure 1b). Treatment with MK-X efficiently decreased the infarction area, while EPO showed no significant protective effect (Figures 1c–e).

### MK-X ameliorates mitochondrial damage under glutamate-induced excitotoxicity by inhibiting calcium overload and oxidative stress

**Figure 2:**
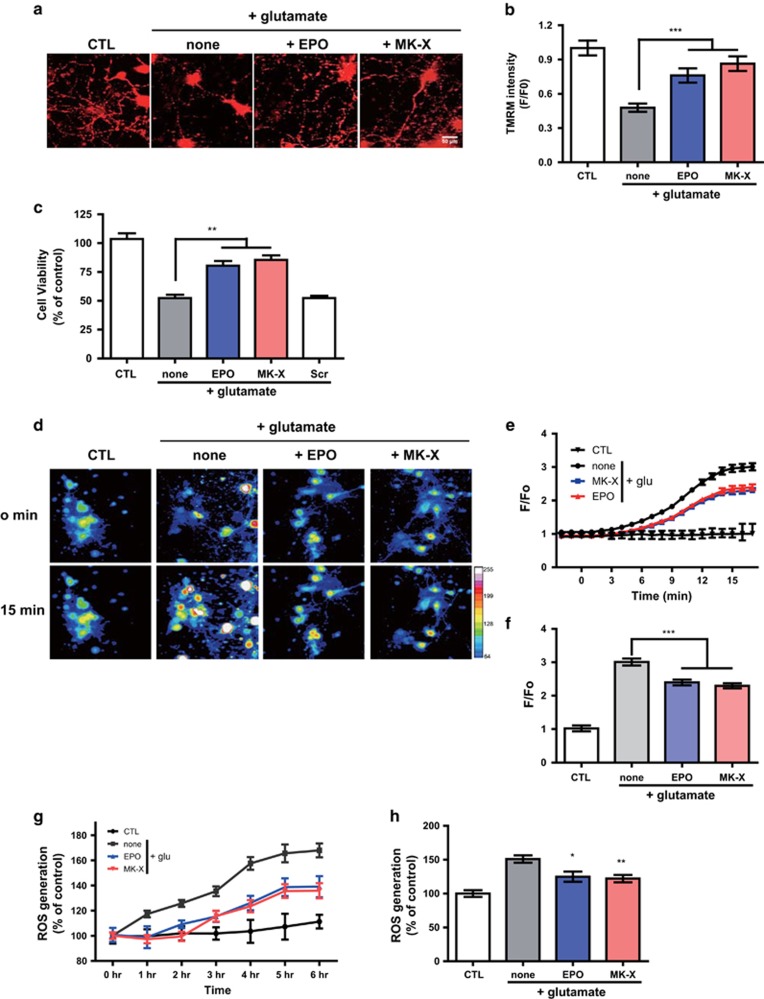
Glutamate-induced excitotoxicity by inhibiting mitochondrial damage mediated by calcium overload and oxidative stress. (**a**) Representative images of TMRM in cultured cortical neurons during glutamate (30 *μ*M) treatment with or without EPO (1 IU) or MK-X (1 pM). The healthy and functioning mitochondria show bright signal. (**b**) Quantitative analysis of TMRM intensity. Cells were incubated with 30 *μ*M glutamate in the presence or absence of EPO (1 IU) or MK-X (1 pM). A culture not treated with glutamate was used as a positive control. (**c**) Neuroprotective effects of various treatments. Experiment was performed as in (**b**) and scrambled peptide (1 pM). Cell viability was assessed by the Calcein-AM assay. (**d**) Representative images of calcium in cultured cortical neurons during glutamate treatment with or without EPO (1 IU) or MK-X (1 pM). (**e**) Time course of intracellular calcium accumulation upon glutamate stress in cultured cortical neurons. Measurements were taken during 15 min after the exposure to glutamate with or without EPO (1 IU) or MK-X (1 pM). Glutamate alone was used as a positive control for intracellular calcium accumulation. (**f**) Quantitative analysis of calcium intensity. Cells were incubated with 30 *μ*M glutamate in the presence or absence of EPO (1 IU) or MK-X (1 pM). A culture not treated with glutamate was used as a negative control. Glutamate alone was used as a positive control for intracellular calcium accumulation. (**g**) Time course of intracellular ROS generation upon glutamate stress in cultured cortical neurons. Measurements were taken at 0, 1, 2, 3, 4, 5 and 6 h after exposure to glutamate with or without EPO (1 IU) or MK-X (1 pM). Glutamate alone was used as a positive control for intracellular ROS generation. (**h**) Quantitative analysis of intracellular ROS generation upon glutamate stress in cultured cortical neurons (6 h). A culture not treated with glutamate was used as a negative control. Glutamate alone was used as a positive control for intracellular ROS generation. Data are means±S.D. from five independent experiments. For statistical analysis, one-way ANOVA was performed, followed by Dunnett’s *post hoc* test. Statistical significance is denoted (*P<0.05, **P<0.01, and ***P<0.001)

To understand the cause of the difference between the effects of MK-X and EPO, we examined the relevant molecular mechanisms *in vitro*. Neurodegeneration caused by ischemic stroke and reperfusion mainly results from mitochondrial dysfunction caused by glutamate-induced excitotoxicity.^26^ MK-X and EPO suppressed the glutamate-induced fragmentation and depolarization of mitochondria which resulted in morphological and functional defects, respectively. Thus, we compared the protective effects of MK-X and EPO against glutamate-induced excitotoxicity in cultured neurons. Both MK-X and EPO efficiently suppressed glutamate-induced dissipation of the mitochondrial membrane potential (Figures 2a and b) and neuronal death (Figure 2c). These data indicate that both MK-X and EPO ameliorate mitochondrial damage caused by glutamate that results in neuronal death. Because calcium overload and oxidative stress induced by ROS cause mitochondrial damage in glutamate-induced excitotoxicity,^27, 28^ We examined whether MK-X could affect this stress-related processes similarly to the case of EPO. We have shown that both MK-X and EPO significantly reduced ROS generation upon glutamate treatment (Figures 2g and h). Notably, glutamate-induced rise in intracellular calcium was also inhibited by MK-X and EPO (Figures 2e and f), implying that MK-X and EPO may regulate NMDAR activity. These data indicate that the neuroprotective effect of MK-X against glutamate-induced excitotoxicity can be explained by its ability to reduce mitochondrial damage mediated by calcium overload and oxidative stress.

### MK-X inhibits caspase-dependent and -independent cell death pathways under glutamate-induced excitotoxicity

**Figure 3:**
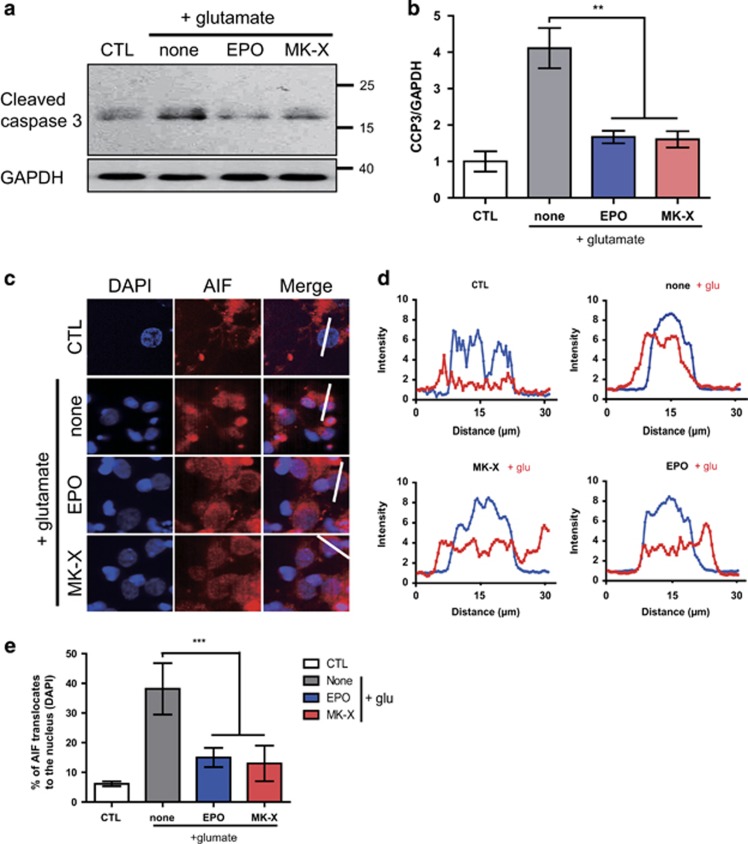
MK-X and EPO prevent activation of both caspase-dependent and -independent cell death pathways under glutamate-induced excitotoxicity. (**a**) After 24 h incubation, MK-X and EPO suppress the cleaved caspase 3 (CCP3). Activation of caspase 3 was measured using antibody against CCP3. Equal loading of samples was confirmed by monitoring GAPDH. (**b**) Quantification of CCP3. Quantification of protein levels was determined by densitometrical analysis using the ImageJ program. (**c**) After 24 h incubation, MK-X and EPO also prevent nuclear translocation of AIF. AIF translocation into the nuclei was examined by immunocytochemistry using antibody against AIF (red). DAPI was used to stain the nuclei (blue). MK-X and EPO showed slight reduction in nuclear translocation of AIF. (**d**) Histogram for translocation of AIF to the DAPI-labeled nuclear region. Red line: AIF, Blue line is DAPI. Total distance of analysis was 30 *μ*m. (**e**) Quantification of AIF translocation. A culture not treated with glutamate was used as a negative control. Glutamate alone was used as a positive control for AIF translocation with the DAPI signal. Data are means±S.D. from five independent experiments. For statistical analysis, one-way ANOVA was performed, followed by Dunnett’s *post hoc* test. Statistical significance is denoted (**P<0.01, and ***P<0.001)

Mitochondrial injury under glutamate-induced excitotoxicity results in a release of cytochrome *c* and AIF from the mitochondrial intermembrane space, which leads to activation of caspase-dependent and caspase-independent cell death pathways.^29, 30^ We found that glutamate treatment increased cleavage of caspase-3, which is the final executor of caspase-dependent cell death,^31^ and that MK-X efficiently reduced caspase-3 cleavage, similar to EPO (Figures 3a and b). Nuclear translocation of AIF, which is an initiator of caspase-independent cell death, was reduced by glutamate and was blocked by MK-X or EPO (Figures 3c and d). A large portion of AIF merge to the nucleus in neurons following glutamate-induced excitotoxicity, whereas treatment of MK-X reduced the translocation of AIF like EPO (Figure 3e). These results indicate that MK-X prevents both caspase-dependent and -independent cell death pathways activated by glutamate.

### MK-X activates multiple secondary signaling pathways under oxidative stress which link with EPO activation

**Figure 4:**
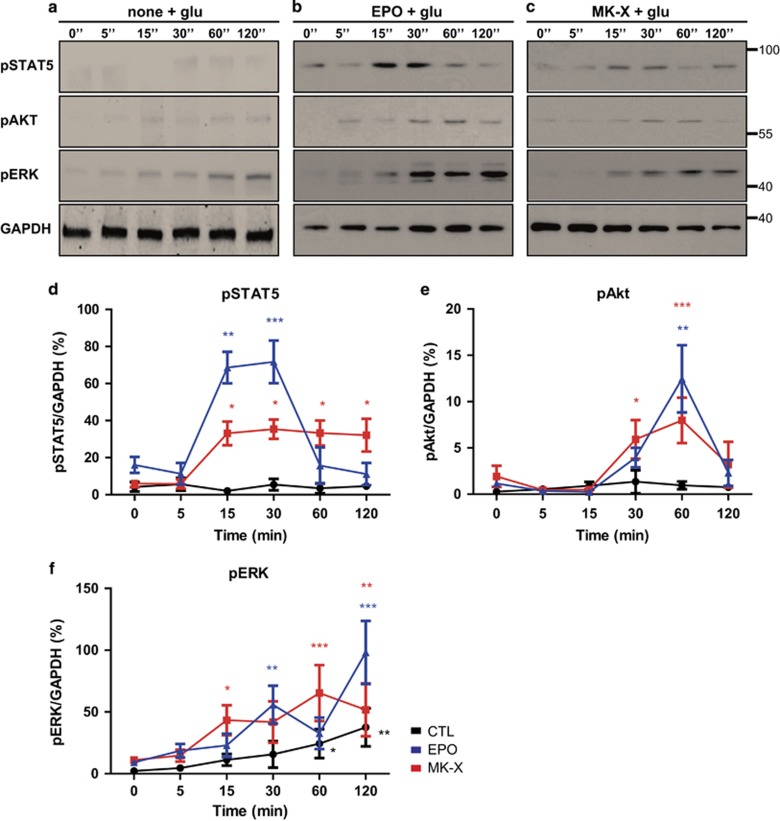
MK-X and EPO activate pathways downstream of EPO-receptor with different kinetics. Cells were treated with 30 *μ*M glutamate for a specified period of time (0, 5, 15, 30, 60, or 120 min) in the presence or absence of MK-X (1 pM) or EPO (1 IU). Representative blots are shown in (**a**–**c**). Equal loading was confirmed by monitoring the GAPDH protein level. The levels of phosphorylated STAT5 (**d**), Akt (**e**), and ERK (**f**) were quantified by stereological analysis using the ImageJ program. Data are means±S.D.from five independent experiments. For statistical analysis, one-way ANOVA was performed, followed by Dunnett’s *post hoc* test. Statistical significance is denoted (*P<0.05, **P<0.01, and ***P<0.001)

Activation of the EPO–EPO-receptors complex turns on the JAK2 signaling pathway, resulting in canonical activation of STAT5 by phosphorylation.^32, 33^ The JAK2 pathway also activates multiple secondary signaling pathways, including MAPKs and PI3K, in frequently studied cell types such as erythrocytes, neurons, astrocytes.^34, 35^ Thus, we examined the downstream signaling pathways that might contribute to the neuroprotective effect of MK-X. Similar to EPO, MK-X increased phosphorylation of STAT5, Akt, and ERK1/2 (Figures 4a–c), although there were some differences in the kinetics of activation. Notably, MK-X induced sustained activation of STAT5, while EPO-induced activation of STAT5 signaling was transient (Figures 4a and d). In contrast, peak levels of phosphorylated Akt and ERK1/2 were lower with MK-X than with EPO (Figures 4a–c). These data indicate that MK-X mimics the neuroprotective effect of EPO, although they exhibit somewhat differential kinetics.

### Neuroprotective effect of MK-X against glutamate-induced excitotoxicity is mediated by JAK2 activation

**Figure 5:**
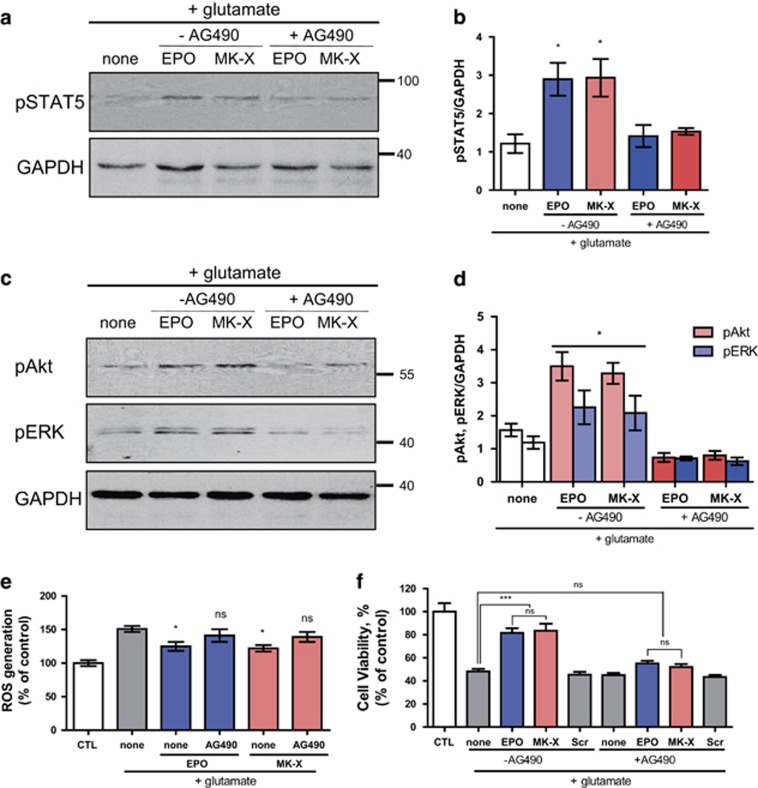
Effects of both MK-X and EPO are mediated by the JAK2 signaling pathway. (**a**) In the presence of 30 *μ*M glutamate, cells were incubated with EPO (1 IU) or MK-X (1 pM). To block the activation of JAK2, cells were pre-incubated with 20 *μ*M AG490. AG490 completely blocked the MK-X and EPO-induced activation of STAT5 (30 min) (**a**), Akt, and ERK (1 h) under oxidative stress condition. (**c**) Quantitative analysis of five independent experiments was performed (**b**,**d**). (**e**) Quantification of intracellular ROS generation upon glutamate stress in cultured cortical neurons. Measurements were taken 6 h after exposure to glutamate with or without EPO (1 IU) or MK-X (1 pM). Glutamate treatment alone was used as a positive control for intracellular ROS generation. (**f**) Neuroprotective effects of various treatments. In the presence of 30 *μ*M glutamate, cells were incubated with EPO (1 IU) or MK-X (1 pM). A culture not treated with glutamate was used as a positive control. Cell viability was assessed by the Calcein-AM assay. Data are means±S.D. from five independent experiments. For statistical analysis, one-way ANOVA was performed, followed by Dunnett’s *post hoc* test. Statistical significance is denoted (*P<0.05, **P<0.01, and ***P<0.001)

To rule out an off-target effect of MK-X, we examined whether its neuroprotective effect is mediated by the EPO-receptor activated JAK2 signaling pathway. We treated cells with a JAK2-STAT5-specific inhibitor, AG490, and monitored canonical activation of STAT5. As expected, AG490 blocked the increase in phosphorylation of STAT5 by MK-X and EPO (Figures 5a and b) and activation of secondary downstream effectors including ERK1/2 and Akt pathways (Figures 5c and d). Furthermore, the effects of MK-X and EPO on neuronal death (Figure 5f) and ROS generation (Figure 5e) were blunted by AG490. Collectively, these data demonstrate that the neuroprotective effect of MK-X is mediated by the JAK2 signaling pathway, similar to that of EPO, and is not caused by off-target stimulation.

### ERK1/2 and Akt signaling pathways are involved in the neuroprotective effect of MK-X

**Figure 6:**
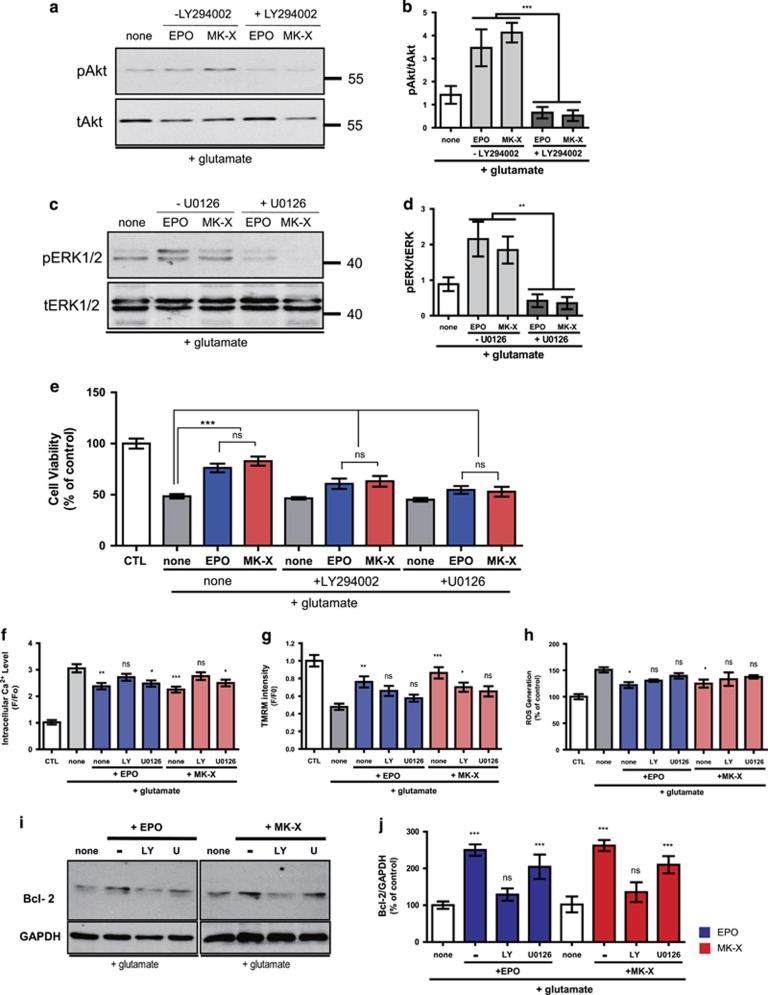
AKT and ERK1/2 contribute differently to the neuroprotective effects of MK-X and EPO. (**a**) MK-X- and EPO-induced Erk1/2 and Akt activation in the presence or absence of U0126 (1 h) under oxidative stress condition. MK-X and EPO increased pErk1/2 levels. These effects were completely blocked by pre-incubation with U0126. (**b**) Quantitative analysis Erk1/2 and Akt activation in five independent experiments. (**c**) MK-X- and EPO-induced Akt activation in the presence or absence of LY294002. MK-X and EPO increased pAkt levels. These effects were completely blocked by pre-incubation with LY294002. (**d**) Quantitative analysis of Akt activation in five independent experiments. (**e**) Neuroprotective effects of treatments with specific inhibitors. In the presence of 30 *μ*M glutamate, cells were incubated with EPO (1 IU) or MK-X (1 pM). A culture not treated with glutamate was used as a positive control. To block the activation of ERK1/2 and Akt, cells were incubated with 20 *μ*M U0126 and50 *μ*M LY294002, respectively. Cell viability was assessed by the Calcein-AM assay. Calcium accumulation (**f**), TMRM staining (**g**), and ROS generation (**h**) were observed in cells treated with EPO (1 IU) or MK-X (1 pM) and a specific inhibitor (20 *μ*M U0126 or 50 *μ*M LY294002). (**i**) Immunoblots for Bcl-2 and GAPDH after incubation for 12 h with EPO (1 IU) or MK-X (1 pM) and a specific inhibitor (20 *μ*M U0126 or 50 *μ*M LY294002). (**j**) Protein levels were quantified by stereological analysis using the ImageJ program. Equal loading was confirmed by monitoring the GAPDH protein level. Data are means±S.D. from five independent experiments. For statistical analysis, one-way ANOVA was performed, followed by Dunnett’s *post hoc* test. Statistical significance is denoted (*P<0.05, **P<0.01, and ***P<0.001)

Previous studies have demonstrated that Akt and ERK1/2 signaling pathways are critical for the neuroprotective effect of EPO under oxidative stress.^36^ To examine the involvement of ERK1/2 and Akt pathways in neuroprotection by MK-X, we pretreated cells with LY294002 and U0126, which are specific inhibitors of PI3K and MEK, respectively. LY294002 and U0126 efficiently reduced phosphorylation of Akt and ERK1/2 increased by MK-X or EPO (Figures 6a–d). The protective effects of MK-X and EPO against glutamate-induced neuronal death were significantly reduced by LY294002 or U0126 (Figure 6e). Consistently, the recovery of the mitochondrial membrane potential by MK-X and EPO was also inhibited by LY294002 or U0126 (Figure 6f), as was the decrease in ROS generation (Figure 6g). These data indicate that EPO- and MK-X-induced neuroprotection against oxidative stress is mediated by the activation of ERK1/2 and Akt signaling pathways. On the other hand, the inhibition of calcium overload by EPO and MK-X was suppressed by LY294002 but not by U0126 (Figure 6h). In addition, treatment with EPO or MK-X increased the expression of Bcl-2, an anti-apoptotic protein that promotes neuronal survival and is involved in EPO-mediated protective function^37^; the increase in Bcl-2 expression was also inhibited by LY294002 but not by U0126 (Figures 6i and j). These data indicate that Akt and ERK1/2 have differential contribution to the neuroprotective effect of MK-X and EPO.

### MK-X and EPO similarly affect gene expression under glutamate-induced excitotoxicity

**Figure 7:**
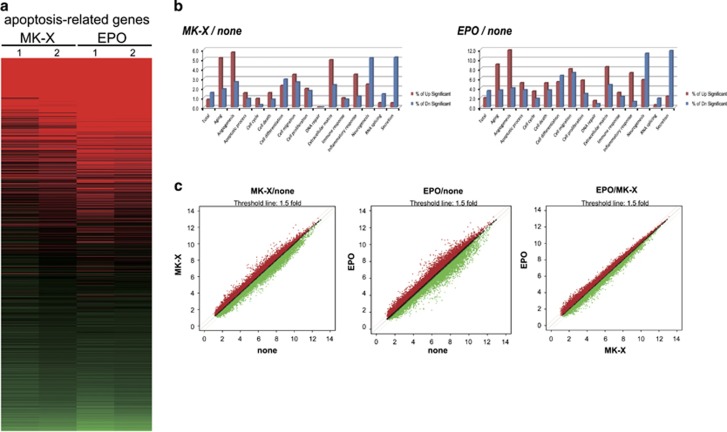
Microarray analysis of genes contributing to the neuroprotective effects of MK-X and EPO. (**a**) Heat-map visualization of 2488 probes for cell death-associated genes in MK-X (lanes 1, 2)- or EPO (lanes 3, 4)-treated cultured cortical neurons under glutamate-induced excitotoxicity. (**b**) Similarity of gene expression profiles in cells treated with EPO (1 IU) and MK-X (1 pM). (**c**) Scatter plots comparing log-ratio differential expression values from each microarray platform (Affymetrix Rat 2.0 ST array)

To identify the genes associated with MK-X and EPO treatments, we compared gene expression in cultured cortical neurons treated with glutamate in the presence or absence of MK-X or EPO (Figures 7a and b). In our results, we compared acquired microarray expression pattern with RNA molecule, including the anti-apoptosis-related gene expression pattern (Supplementary Figures S1A–C). Interestingly, treatment with MK-X efficiently induced the expression of anti-apoptotic pathway-related genes, and its effect was similar to that of EPO (Figure 7c,Supplementary Figure S1D). These data indicate that the neuroprotective effect of MK-X is accompanied by changes in the expression of various genes, and that gene expression profiles in glutamate-stressed neurons treated with MK-X and EPO are similar.

### MK-X penetrates into the brain across the BBB more rapidly than does EPO

**Figure 8:**
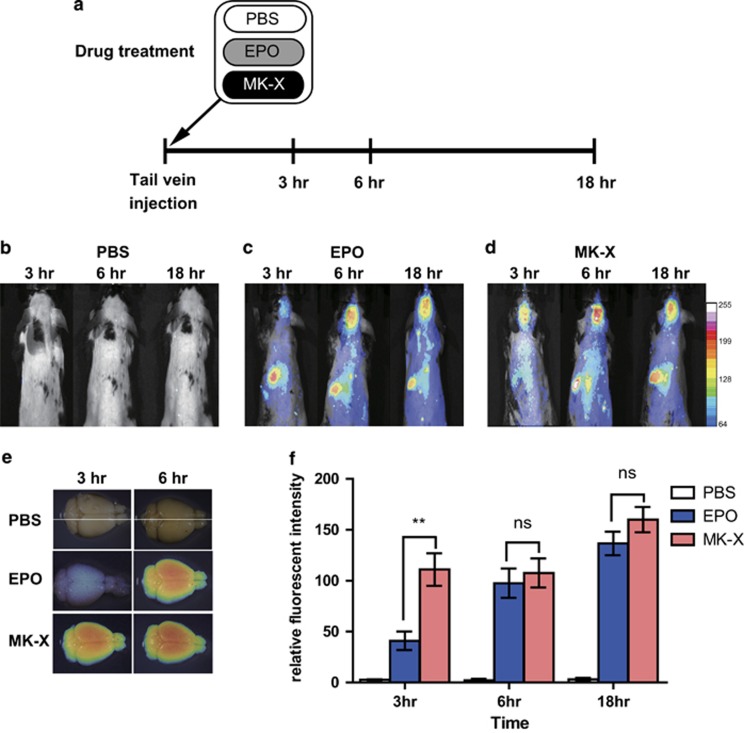
Distribution of QDs in mouse brains following the intravenous administration of QDs coated with MK-X or EPO, or negative control with PBS. (**a**) Experimental scheme for the assessment of BBB penetration of MK-X and EPO treatments. (**b**–**d**) Representative *in vivo* images of fluorescent QD655 under each condition. (**e**) Representative images with whole brain after perfusion (3, 6 h). (**f**) Quantification of average fluorescence intensity in rat brains (*n*=3). For statistical analysis, one-way ANOVA was performed, followed by Dunnett’s *post hoc* test. Statistical significance is denoted (**P<0.01)

During reperfusion, excitotoxicity is a major factor involved in neuronal death and appears within several hours.^38, 39^ Therefore, therapeutic time is critical for the effectiveness of anti-stroke treatments after reperfusion.^40^ To be effective against CNS diseases, drugs have to reach the brain by crossing the BBB. To explain why *in vivo* neuroprotective effect of MK-X is stronger than that of EPO, we assessed penetration of MK-X and EPO across the BBB. We administered MK-X- or EPO-conjugated quantum dots (QDs) via tail vein injection and monitored MK-X and EPO penetration into the brain at 3, 6, and 18 h after the injection (Figure 8a). Strikingly, we found that the level of MK-X in the brain was significantly higher than that of EPO at 3 h (Figure 8b), while EPO exhibited slower time-dependent accumulation (Figures 8d–f). These results indicate that MK-X penetrates into the brain across the BBB more rapidly than does EPO. Taken together, our data suggest that MK-X has a potential as a drug for acute treatment after ischemic stroke.

## Discussion

We have previously proposed a novel EPO-derived short peptide, MK-X, which has a neuroprotective effect against oxidative stress without adverse side effects characteristic of EPO such as unwanted cell proliferation.^41^ In this study, we examined the neuroprotective effect of MK-X in comparison with that of EPO against glutamate-induced excitotoxicity induced *in vitro* in cultured neurons and against brain injury in an *in vivo* stroke model.

In the current study, we attempted to increase the therapeutic time window for a drug because the infrastructure to treat patients quickly is not established. In case of EPO administration, multiple daily doses are more effective than a single dose.^42^ However, administration of EPO may induce a number of serious adverse effects including a higher mortality rate and higher risk of thromboembolic complications.^43^ Because of the side effects of EPO, its dose approved for use in patients is at the lower limit of the single-dose regimen that has been effective in experimental ischemic models. Therefore, the opportunity to rapidly treat patients with a single dose within the ‘golden hour’ before extensive neuronal loss occurs is considered extremely important to eliminate side effects.^44, 45^ Another difficulty is that the appropriate single dose needs to be tested empirically. To explore the potential of MK-X to be developed as a drug for stroke treatment, we subjected rats to MCAO for 2 h followed by reperfusion for 24 h with single injection of saline, EPO, or MK-X. Saline treatment resulted in extensive infarction in the cerebral cortical and subcortical areas, which was detectable over a series of brain sections. Surprisingly, while EPO treatment for 2 h after MCAO failed to diminish ischemic lesion volume, MK-X treatment significantly decreased infarct volume under the same conditions. To explain the difference in the effects of MK-X and EPO, we first confirmed their effects on survival-related mechanisms in cultured neurons. The neuroprotective effect of EPO under glutamate-induced excitotoxicity appears to be mediated by inhibition of production and/or release of glutamate and ROS, and attenuation of cell death.^34, 37^ Our results show that both MK-X and EPO decrease glutamate-induced elevation of ROS and inhibit cleavage of caspase-3 and nuclear translocation of AIF. A previous study showed that EPO reduces exocytotic glutamate release by glutamate treatment and thus decreases the total concentration of glutamate, which is the major excitatory neurotransmitter; and mitigates excitotoxicity from its earliest stage. The effects of EPO on calcium signaling in excitotoxicity are not yet known, but EPO has well-characterized effects on the Ca^2+^ homeostasis of neuronal cells.^46^ Additionally, EPO might regulate neuronal function and viability by modulating intracellular calcium levels through a voltage-independent ion channel permeable to calcium.^47^ Recent studies have demonstrated that EPO modulates TRP channels through a mechanism requiring the activation of phospholipase C and inositol 1,4,5-triphosphate receptor.^48, 49^ Our results show that both MK-X and EPO attenuate the glutamate-induced increase in calcium influx and concomitant excitotoxicity in cultured cortical neurons. Besides indirect neuroprotection via calcium modulation, numerous lines of evidence support a direct anti-excitotoxic effect of EPO. MK-X and EPO both inhibited ROS generation and dissipation of the mitochondrial membrane potential and displayed quantitatively similar survival effects. EPO is well known to have antiapoptotic effects by inducing synthesis of antioxidants; this property is also related to its ability to maintain the mitochondrial membrane potential.^50^ Similarly, MK-X may function as an antioxidant and have an anti-excitotoxic effect under oxidative stress.

Cell death after cerebral ischemia was considered to be exclusively necrotic in nature, but research over the past decade has revealed that after a stroke, many neurons in the ischemic penumbra undergo apoptosis.^51^ To characterize apoptosis caused by glutamate-induced excitotoxicity, we confirmed the effect on increased intracellular calcium levels, mitochondrial membrane potential and the proapoptotic proteins caspase 3 and AIF.^52^ Consistent with their antioxidant effects, both MK-X and EPO decreased activation of the apoptotic cascade after induction of excitotoxicity. Taken together, our data suggest that MK-X-mediated neuroprotection under oxidative stress is similar to that conferred by EPO.

MK-X, distinct from EPO, has induced different kinetics of signaling pathways. The different activation mechanisms of MK-X can be explained by the different activation of receptors. MK-X, distinct from EPO, may just activate a different EPO receptor or may just fail to activate the ‘homodimeric EPOR’, while in contrast, EPO would activate homodimeric and other EPO receptors. To explain the specific mechanisms triggered under oxidative stress by EPO and MK-X in rat cortical neurons, we analyzed three signaling pathways induced by EPO-receptors activation including JAK/STAT, MAPK, and PI3K/Akt. Among these pathways, STAT5 signaling is involved in neuroprotective mechanisms of EPO though parallel pathways (such as ERK1/2 and PI3K signaling) are typically activated simultaneously.^8, 37, 53, 54^ Our findings suggest that MK-X may exert neuroprotective effects via long-lasting ERK1/2 and PI3K activation, similar to the mechanism reported for EPO.^55^ Our inhibitor studies suggest that EPO and MK-X protect neurons mainly through both ERK and Akt pathways, but in different ways. The PI3K/Akt signaling pathway act mainly via Ca^2+^ modulation, whereas the MAPK/ERK pathway promotes functional recovery of mitochondria from excitotoxicity. Although these pathways use different protective mechanisms, they have similar effects on cell viability. Our current study shows that both ERK and Akt pathways are essential for neuroprotection, and inhibition of either of them prevented complete inhibition of oxidative stress-induced apoptosis. Similarity of the effects of MK-X and EPO on protective signaling pathways reflects the fact that they share the same pathway that involves EPO-receptor. In our study, MK-X and EPO also shared similar gene expression profiles.

Although EPO and MK-X displayed similar protective effects *in vitro*, MK-X showed better protective effects than EPO in our experimental cerebral ischemic stroke model (Figure 1). To account for this difference in the protective effects between *in vivo* and *in vitro*, we focused on the time profile of the transport of MK-X and EPO across the BBB. Studies of several brain diseases have demonstrated that peripherally administered EPO crosses the BBB and alters neuronal survival and functional recovery.^8, 56^ One possible explanation of the functional gap between MK-X and EPO could be that a small peptide has potential advantages in crossing the BBB.^57^ If so, because acute brain ischemia is strongly time-dependent, the protective effect of MK-X may be stronger than that of EPO under our experimental conditions. Although proteins and peptides normally do not cross the BBB without a receptor-mediated translocation system, some studies have suggested that peptides and small molecules may use specific transporters expressed on the luminal and the basolateral sides of endothelial cells, which allows them to be transported across the BBB into the brain.^8, 58^ Indeed, our results show that MK-X penetrates into the brain across the BBB more rapidly than does EPO. Previous studies found that intravenous medications need to be administered to stroke victims within the so-called ‘golden hour’, that is, within 3 h (up to 4.5 h in certain patients), during which the patients have the best chance to survive and avoid debilitating, long-term neurological damage.^59, 60^ Compared to EPO, MK-X could have a higher chance of providing protection in damaged brain in stroke patients. Therefore, we suggest MK-X as a promising neuroprotective therapeutic agent that can be expected to reach future clinical pilot trials.

## Materials and methods

### Culture of rat cortical neurons and chemical agents

Embryonic cerebral cortices were obtained from Sprague–Dawley rats on embryonic day 17. Briefly, cerebral cortices were physically and chemically dissociated with 0.25% trypsin–EDTA (Gibco, Rockville, MD, USA), and then the dissociated cells were plated on plates or cover-slips coated by 50 *μ*g/ml poly-L-lysine (Sigma, St. Louis, MO, USA) at a density of 2 × 10^5^ cells/cm^2^ in plating medium composed of B27 supplement (Gibco), 200 mM L-glutamine (Gibco), 25 *μ*M glutamic acid (Sigma) and penicillin/streptomycin (Gibco) in neurobasal medium (Gibco). The glutamate (25 *μ*M) should be included in media only for the plating stage. The medium was changed to a fresh medium without glutamate at 3 days *in vitro*. Glutamic acid (30 *μ*M; Sigma) was added to induce excitotoxicity. The MK-X peptide (ISGLRSLTTLLRALGAQKELM) and scrambled peptide (AMELSGRTLGLILKLRQSATL) were prepared as previously reported.^23^

### Cell viability assay

To examine glutamate-induced excitotoxicity, the viability of MK-X and EPO was observed after 24 h. In the presence of 30 *μ*M glutamate, cells were co-incubated with EPO (1 IU/ml) and MK-X (1 pM), respectively. Cell viability was determined by using Calcein-AM (#C3100MP, (Invitrogen, Carlsbad, CA, USA)). Cultured rat cortical neurons plated on 96-well plates (2.5 × 10^4^ cells per well) were incubated with Calcein-AM (3 *μ*M) for 30 min after various treatments. The intensity of the Calcein-AM signal (excitation, 485 nm; emission 535 nm) was measured by using a microplate reader (SpectraMax Plus 384 Microplate Reader, Molecular Devices, Sunnyvale, CA, USA).

### Measurement of calcium, mitochondrial membrane potential and ROS

To characterize glutamate-induced intracellular calcium increases upon glutamate treatment, calcium levels were observed for 15 min in presence of MK-X(1 pM) and EPO (1 IU/ml). To quantify intracellular calcium levels, cultured cortical neurons were stained with 3 *μ*M Fluo-3 AM (Molecular Probes, Eugene, OR, USA) in phosphate-buffered saline (PBS) at 37 °C for 30 min. After several washes using PBS, the medium was replaced with transparent DMEM/F-12 containing no phenol red (Invitrogen) and Fluo-3 AM. Cells (>100 cells) were visualized under a Zeiss LSM 7 Live microscope (Carl Zeiss) using a  ×  20 objective. Collected images were analyzed using ImageJ software.

To examine the glutamate-induced change in mitochondrial membrane potential, mitochondrial membrane potential with administration of MK-X and EPO was observed for 4 h. Cells were co-incubated with EPO (1 IU/ml) and MK-X (1 pM) in the presence of 30 *μ*M glutamate, respectively. Mitochondrial membrane potential was determined by adding 10 nM TMRM (Molecular Probes) to culture medium for 20 min and then cells (>100 cells) were visualized under a Zeiss LSM 7 Live microscope (Carl Zeiss) using a  ×  20 objective. Collected images were analyzed using ImageJ software. To examine the glutamate-induced ROS generation, ROS level of MK-X and EPO was observed for 6 h. Cells were co-incubated with EPO (1 IU/ml) and MK-X (1 pM) in the presence of 30 *μ*M glutamate, respectively. To quantify intracellular ROS, cultured cortical neurons were stained with 5 *μ*M CM-H2DCFDA (Molecular Probes) in PBS for 30 min. After several washes in PBS, the cells were resuspended in culture medium without CM-H2DCFDA for 2 h. Fluorescence intensity of CM-H2DCFDA was measured in the microplate reader (excitation, 485 nm; emission, 535 nm).

### Immunoblotting

Cells were lysed in lysis buffer (50 mM Tris, pH 7.4, 150 mM NaCl, 1% Triton X-100, 1% deoxycholic acid, and 0.1% SDS) on ice for 30 min. Lysate (10 *μ*g of protein) was loaded on SDS-PAGE and proteins were transferred onto polyvinylidene difluoride membranes. The membranes were blocked with 1% skimmed milk for 1 h and probed with primary antibody for 1–2 h at room temperature. Primary antibodies against phospho-Akt (Cell Signaling Technology, Beverly, MA, USA), phospho-ERK1/2 (Cell Signaling), phospho-STAT5 (Cell Signaling), phospho-p38 (Cell Signaling Technology), and GAPDH (Chemicon, Temecula, CA, USA) were used at 1:1000 dilution. After several washes, membranes were incubated with the following secondary antibodies at a 1:5000 dilution: horseradish peroxidase-conjugated goat anti-rabbit IgG to detect phospho-Akt, phospho-ERK1/2, phospho-STAT5, or anti-mouse IgG to detect GAPDH. Proteins were visualized using an enhanced chemiluminescence substrate kit (Pierce, Rockford, IL, USA). Blots were scanned and quantified by using the ImageJ program (NIH, Bethesda, MD, USA).

### Experimental cerebral ischemic stroke

We induced reversible ischemia (occlusion–reperfusion) to develop a transient MCAO model. Briefly, 31 animals were randomly divided into experimental (*n*=21 (EPO, 10; MK-X, 11)) or sham (*n*=10) groups and anesthetized by inhalation of isoflurane in medical-grade oxygen. The sternocleidomastoid muscle was dissected to expose the right common carotid artery, external carotid artery, and internal carotid artery. The arteries were separated from the surrounding nerves and connective tissue. The external carotid artery was coagulated for 2 h with filament insertion. At the end of the ischemic period, the animals were re-anesthetized and the filament was gently retracted to allow reperfusion of the ischemic region. Animals received a single injection of MK-X (3.6 *μ*g/kg in 0.3 ml of saline, i.p.), EPO (3000 units/kg in 0.3 ml of saline, i.p.), or saline (0.3 ml of saline, i.p.) at the time of reperfusion. Animals from each group were killed 24 h after reperfusion, and their brains were collected for appropriate histochemical staining to assess the neuroprotective effects of MK-X or EPO.

### Measurement of infarct volume

The Animal Care and Use Committee of DGIST approved all animal protocols. After 1 week of acclimatization, each group of C57/BL mice underwent occlusion–reperfusion and were killed under anesthesia 24 h later. The brains were rapidly removed. Coronal sections were cut into 2-mm-thick slices and stained with standard 2% TTC for 20 min at 37 °C. The pale infarcted areas and areas of the non-infarcted hemispheres were digitally analyzed using NIH ImageJ software. The area of infarction from each slide was added and presented as percentage of the volume of the non-infarcted hemisphere.

### Preparation of a peptide–quantum dot conjugate

To analyze the capability of peptide-directed transport through the BBB, the EPO and peptides were incorporated into the surface of QDs (Qdot 655 ITK Carboxyl, Life Technologies, Carlsbad, CA, USA) according to the manufacturer’s instructions. Briefly, 500 *μ*l of 1 *μ*M target peptide was added to 500 *μ*l of 1 *μ*M QD solution in 10 mM borate buffer (pH 7.4) and incubated with stirring. We then prepared 1-ethyl-3-(3-dimethylaminopropyl) carbodiimide solution (10 mg/ml) just before use and added it to the peptide–QD mixture. The mixture was stirred gently for 1 h at room temperature for conjugation. The conjugate solution was filtered through a 0.2 *μ*m syringe filter to remove any large aggregates, transferred to a clean centrifugal tube and centrifuged at 10 000 × *g* for 5 min. The precipitate was resuspended in PBS and washed three times to remove any excess unbound peptides. Finally, the peptide–QD conjugates were resuspended in 500 *μ*l PBS.

### *In vivo* assessment of MK-X penetration into the brain with QD nanoparticles

After 1 week of acclimatization, each group of C57/BL mice was intravenously injected with 100 *μ*l protein–QD conjugate solution (~1 *μ*M QD). *In vivo* optical imaging was conducted by scanning the brain (excitation, 470 nm; emission, 590 nm) under anesthesia using Optix exPlore (Advanced Research Technologies Inc., Montreal, Canada). Fluorescence was observed using IVIS Spectrum (IVISSPE; PerkinElmer, Inc., Waltham, MA, USA) for Qdot655. Average total fluorescence intensity was provided by the IVIS instrument software.

### Statistical analysis

All data are presented as the mean (±) standard error of the mean (S.E.M.). One-way analysis of variance followed by Dunnett’s *post-hoc* test was used and the criterion for statistical significance was set at *P*<0.05.
