# Supplementary figures and images for: The erythropoietin-derived peptide MK-X and erythropoietin have neuroprotective effects against ischemic brain damage
Source: Cell Death Dis. 2017 Aug 17;8(8):e3003–. doi: 10.1038/cddis.2017.381 (PMC5596568; doi:10.1038/cddis.2017.381)

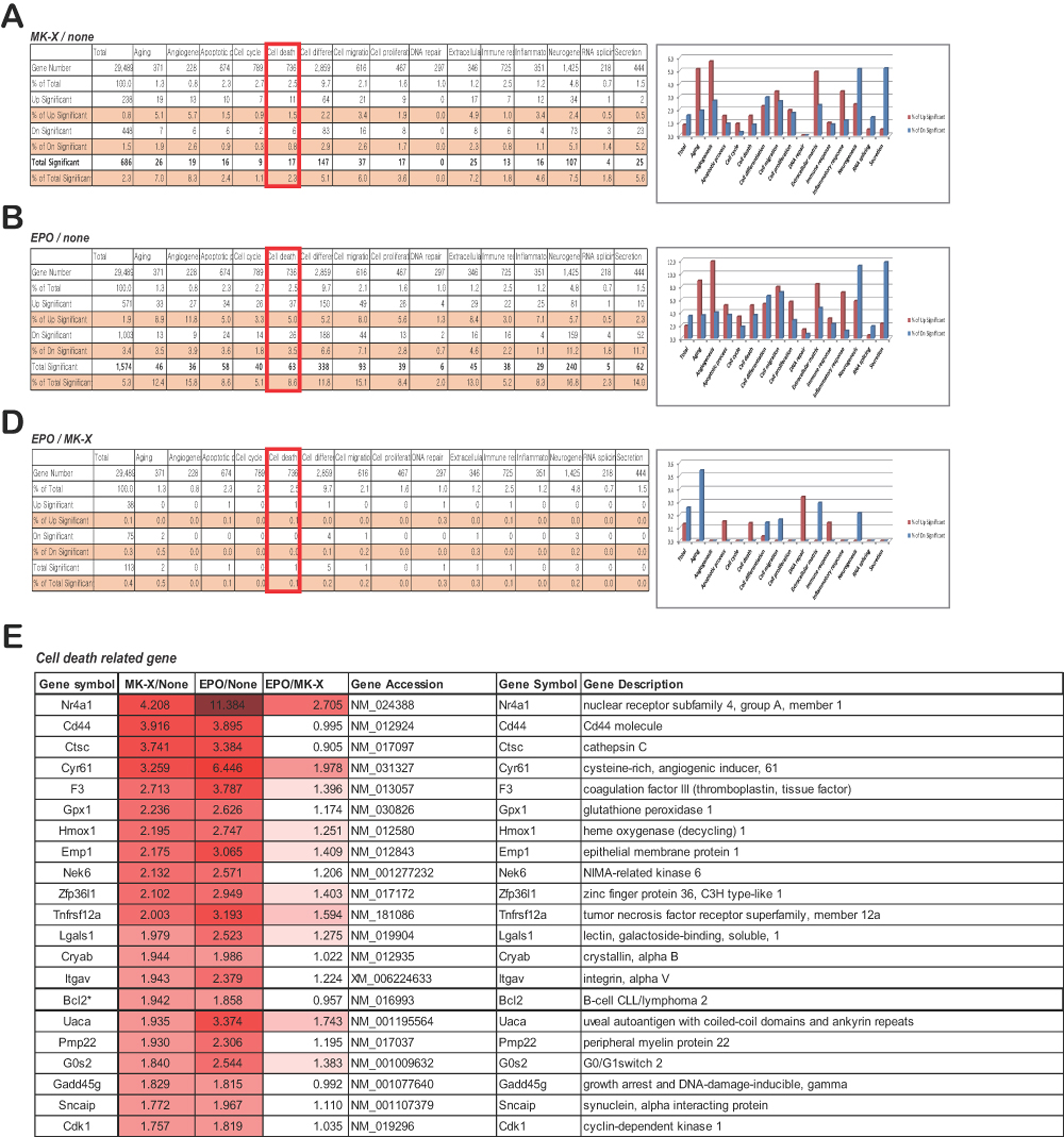

Supplement: Supplementary Figure 1 [file cddis2017381x1.tif]
