# Supplementary material for: The erythropoietin-derived peptide MK-X and erythropoietin have neuroprotective effects against ischemic brain damage
Source: Cell Death Dis. 2017 Aug 17;8(8):e3003–. doi: 10.1038/cddis.2017.381 (PMC5596568; doi:10.1038/cddis.2017.381)
Supplement: Supplementary Information [file cddis2017381x2.pdf]

Supplementary Information:

The erythropoietin-derived peptide MK-X and erythropoietin have neuroprotective effects against ischemic brain damage

Running title: MK-X has the potential to become a novel drug for protection from brain injury caused by ischemic stroke

Seung-Jun Yoo[1], Bongki Cho[1, 2], Deokho Lee[1], Gowoon Son[1], Yeong-Bae Lee[3], Hyung Soo Han[4], Eunjoo Kim[5], Chanil Moon[6], and Cheil Moon[1, 2*]

[1]Department of Brain & Cognitive Sciences, Graduate School, Daegu Gyeongbuk Institute of Science and Technology (DGIST), Daegu 711-873, Republic of Korea. [2] **Convergence Research Advanced Centre for Olfaction, Daegu Gyeungbuk Institute of Science and Technology, Daegu, Korea.** [3]Department of Neurology, Gil Medical Center, Gachon University, Incheon 405-760, Republic of Korea. [4]Department of Physiology, School of Medicine, Kyungpook National University, Daegu 700-422, Republic of Korea. [5]Division of Nano and Energy Convergence Research, Daegu Gyeongbuk Institute of Science and Technology (DGIST), 711-873, Republic of Korea. [6]GemVax, Unjun-ro, Bundang Seongnam-si, Gyeonggi-do,13467, Republic of Korea.

***Correspondence: Cheil Moon, PhD, Department of Brain & Cognitive Sciences, Graduate School, Daegu Gyeungbuk Institute of Science and Technology, 333, Techno Jung-Ang Daero, Hyeonpung-Myeon, Dalseong-Gun, Daegu, 711-873, Korea. E-mail: cmoon@dgist.ac.kr; Tel: +82-53-785-1040; Fax: +82-53-785-6109**

Competing financial interest : There is NO Competing Interest.

Acknowledgements : This work was supported by the Ministry of Science, ICT and Future Planning & DGIST (16-BD-0402, DGIST Convergence Science Center)

Supplementary Fig 1.

A

MK-X / none

	Total	Aging	Angiogene	Apoptotic p	Cell cycle	Cell death	Cell differen	Cell migratio	Cell proliferat	DNA repair	Extracellula	Immune res	Inflammato	Neurogene	RNA splicin	Secretion
Gene Number	29,489	371	228	674	789	736	2,859	616	467	297	346	725	351	1,425	218	444
% of Total	100.0	1.3	0.8	2.3	2.7	2.5	9.7	2.1	1.6	1.0	1.2	2.5	1.2	4.8	0.7	1.5
Up Significant	238	19	13	10	7	11	64	21	9	0	17	7	12	34	1	2
% of Up Significant	0.8	5.1	5.7	1.5	0.9	1.5	2.2	3.4	1.9	0.0	4.9	1.0	3.4	2.4	0.5	0.5
Dn Significant	[illegible]	7	6	6	2	6	83	16	8	0	8	6	1	73	3	23
% of Dn Significant	1.5	1.9	2.6	0.9	0.3	0.8	2.9	2.6	1.7	0.0	2.3	0.8	1.1	5.1	1.4	5.2
**Total Significant**	**686**	**26**	**19**	**16**	**9**	**17**	**147**	**37**	**17**	**0**	**25**	**13**	**16**	**107**	**4**	**25**
% of Total Significant	2.3	7.0	8.3	2.4	1.1	2.3	5.1	6.0	3.6	0.0	7.2	1.8	1.6	7.5	1.8	5.6

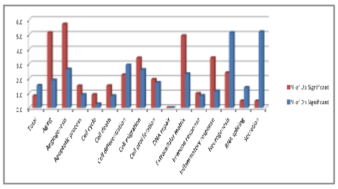

B

EPO / none

	Total	Aging	Angiogene	Apoptotic p	Cell cycle	Cell death	Cell differen	Cell migratio	Cell proliferat	DNA repair	Extracellula	Immune res	Inflammato	Neurogene	RNA splicin	Secretion
Gene Number	29,489	371	228	671	789	736	2,859	616	467	297	346	725	351	1,425	218	441
% of Total	100.0	1.3	0.8	2.3	2.7	2.5	9.7	2.1	1.6	1.0	1.2	2.5	1.2	4.8	0.7	1.5
Up Significant	571	33	27	34	26	37	150	49	26	4	29	22	25	81	1	10
% of Up Significant	1.9	8.9	11.8	5.0	3.3	5.0	5.2	8.0	5.6	1.3	8.4	3.0	7.1	5.7	0.5	2.3
Dn Significant	1,003	13	9	24	14	26	188	44	13	2	16	16	4	159	4	52
% of Dn Significant	3.4	3.5	3.9	3.6	1.8	3.5	6.6	7.1	2.8	0.7	4.6	2.2	1.1	11.2	1.8	11.7
Total Significant	**1,574**	**46**	**36**	**58**	**40**	**63**	**338**	**93**	**39**	**6**	**45**	**38**	**29**	**240**	**5**	**62**
% of Total Significant	5.3	12.4	15.8	8.6	5.1	8.6	11.8	15.1	8.4	2.0	13.0	5.2	8.3	16.8	2.3	14.0

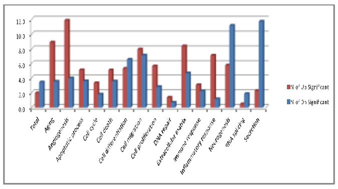

C

EPO / MK-X

	Total	Aging	Angiogene	Apoptotic p	Cell cycle	Cell death	Cell differen	Cell migratio	Cell proliferat	DNA repair	Extracellula	Immune res	Inflammato	Neurogene	RNA splicin	Secretion
Gene Number	29,489	371	228	674	789	736	2,859	616	467	297	346	725	351	1,425	218	444
% of Total	100.0	1.3	0.8	2.3	2.7	2.5	9.7	2.1	1.6	1.0	1.2	2.5	1.2	4.8	0.7	1.5
Up Significant	38	0	0	1	0	1	1	0	0	1	0	1	0	0	0	0
% of Up Significant	0.1	0.0	0.0	0.1	0.0	0.1	0.0	0.0	0.0	0.3	0.0	0.1	0.0	0.0	0.0	0.0
Dn Significant	75	2	0	0	0	0	4	1	0	0	1	0	0	3	0	0
% of Dn Significant	0.3	0.5	0.0	0.0	0.0	0.0	0.1	0.2	0.0	0.0	0.3	0.0	0.0	0.2	0.0	0.0
Total Significant	113	2	0	1	0	1	5	1	0	1	1	1	0	3	0	0
% of Total Significant	0.4	0.5	0.0	0.1	0.0	0.1	0.2	0.2	0.0	0.3	0.3	0.1	0.0	0.2	0.0	0.0

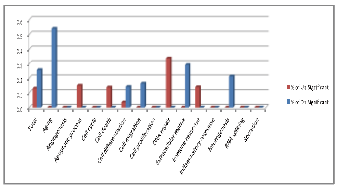

D

Cell death related gene

Gene symbol	MK-X/None	EPO/None	EPO/MK-X	Gene Accession	Gene Symbol	Gene Description
Nr4a1	4.208	11.384	2.705	NM_024388	Nr4a1	nuclear receptor subfamily 4, group A, member 1
Cd44	3.916	3.895	0.995	NM_012924	Cd44	Cd44 molecule
Ctsc	3.741	3.384	0.905	NM_017097	Ctsc	cathepsin C
Cyr61	3.259	6.446	1.978	NM_031327	Cyr61	cysteine-rich, angiogenic inducer, 61
F3	2.713	3.787	1.396	NM_013057	F3	coagulation factor III (thromboplastin, tissue factor)
Gpx1	2.236	2.626	1.174	NM_030826	Gpx1	glutathione peroxidase 1
Hmox1	2.195	2.747	1.251	NM_012580	Hmox1	heme oxygenase (decycling) 1
Emp1	2.175	3.065	1.409	NM_012843	Emp1	epithelial membrane protein 1
Nek6	2.132	2.571	1.206	NM_001277232	Nek6	NIMA-related kinase 6
Zfp36l1	2.102	2.949	1.403	NM_017172	Zfp36l1	zinc finger protein 36, C3H type-like 1
Tnfrsf12a	2.003	3.193	1.594	NM_181086	Tnfrsf12a	tumor necrosis factor receptor superfamily, member 12a
Lgals1	1.979	2.523	1.275	NM_019904	Lgals1	lectin, galactoside-binding, soluble, 1
Cryab	1.944	1.986	1.022	NM_012935	Cryab	crystallin, alpha B
Itgav	1.943	2.379	1.224	XM_006224633	Itgav	integrin, alpha V
Bcl2*	1.942	1.858	0.957	NM_016993	Bcl2	B-cell CLL/lymphoma 2
Uaca	1.935	3.374	1.743	NM_001195564	Uaca	uveal autoantigen with coiled-coil domains and ankyrin repeats
Pmp22	1.930	2.306	1.195	NM_017037	Pmp22	peripheral myelin protein 22
G0s2	1.840	2.544	1.383	NM_001009632	G0s2	G0/G1switch 2
Gadd45g	1.829	1.815	0.992	NM_001077640	Gadd45g	growth arrest and DNA-damage-inducible, gamma
Sncaip	1.772	1.967	1.110	NM_001107379	Sncaip	synuclein, alpha interacting protein
Cdk1	1.757	1.819	1.035	NM_019296	Cdk1	cyclin-dependent kinase 1

Supplementary Fig 1. Treatment with MK-X efficiently induced the expression of anti-apoptotic pathway–related genes, and its effect was similar to that of EPO. (A-B) Analysis of gene expression in the presence of (A) MK-X (1.2 ng/mL) and (B) EPO (0.5 IU/mL), respectively. (C) Direct comparison analysis of gene expression pattern of MK-X and EPO. Cell death associated gene group was shown as red rectangle over the tables. (D) Representative cell death related gene list up-regulated in the presence of MK-X and EPO, respectively.
